# Quantitative Chemical Composition, Anti-Oxidant Activity, and Inhibition of TNF Release by THP-1 Cells Induced by Extracts of *Echinodorus macrophyllus* and *Echinodorus grandiflorus*

**DOI:** 10.3390/antiox12071365

**Published:** 2023-06-29

**Authors:** Marina Pereira Rocha, Lyandra Maciel Cabral da Silva, Laura Paulino Maia Silva, José Hugo de Sousa Gomes, Rodrigo Maia de Pádua, João Aguiar Nogueira Batista, Marcelo Martins Sena, Priscilla Rodrigues Valadares Campana, Fernão Castro Braga

**Affiliations:** 1 Departament of Pharmaceutical Products, Faculty of Pharmacy, Universidade Federal de Minas Gerais, Campus Pampulha, Avenida Antônio Carlos 6627, Belo Horizonte 31270-901, MG, Brazil; procha.marina@gmail.com (M.P.R.); yandramcabral@gmail.com (L.M.C.d.S.); lpmaias@hotmail.com (L.P.M.S.); josehugoufmg@gmail.com (J.H.d.S.G.); rodrigomaiapadua@farmacia.ufmg.br (R.M.d.P.); prvcampana@gmail.com (P.R.V.C.); 2 Departament of Botany and Molecular Biology, Universidade Federal de Minas Gerais, Campus Pampulha, Avenida Antônio Carlos 6627, Belo Horizonte 31270-901, MG, Brazil; janb@icb.ufmg.br; 3 Chemistry Department, Universidade Federal de Minas Gerais, Av. Antônio Carlos 6627, Belo Horizonte 31270-901, MG, Brazil; marcsen@ufmg.br; 4 Instituto Nacional de Ciência e Tecnologia em Bioanalítica (INCT-Bio), Campinas 13083-970, SP, Brazil

**Keywords:** *Echinodorus grandiflorus*, *Echinodorus macrophyllus*, chemical markers, molecular analysis, anti-oxidant activity, inhibition of TNF release, principal component analysis

## Abstract

This study investigated the similarities between *Echinodorus macrophyllus* and *Echinodorus grandiflorus*, plant species that are traditionally used in Brazil to treat rheumatism and arthritis, whose anti-inflammatory effects are supported by scientific evidence. The contents of *cis*- and *trans*-aconitic acid, homoorientin, chicoric acid, swertisin, caffeoyl-feruloyl-tartaric acid, and di-feruloyl-tartaric acid were quantified by UPLC-DAD in various hydroethanolic extracts from the leaves, whereas their anti-oxidant activity and their effect on TNF release by LPS-stimulated THP-1 cells were assessed to evaluate potential anti-inflammatory effects. The 50% and 70% ethanol extracts showed higher concentrations of the analyzed markers in two commercial samples and a cultivated specimen of *E. macrophyllus*, as well as in a commercial lot of *E. grandiflorus*. However, distinguishing between the species based on marker concentrations was not feasible. The 50% and 70% ethanol extracts also exhibited higher biological activity, yet they did not allow differentiation between the species, indicating similar chemical composition and biological effects. Principal component analysis highlighted comparable chemical composition and biological activity among the commercial samples of *E. macrophyllus*, while successfully distinguishing the cultivated specimen from the commercial lots. In summary, no differences were observed between the two species in terms of the evaluated chemical markers and biological activities.

## 1. Introduction

*Echinodorus macrophyllus* (Kunth) Micheli (syn. *Aquarius macrophyllus* (Kunth) Christenh. & Byng., *Alisma macrophyllum* Kunth) and *Echinodorus grandiflorus* (Cham & Schltdl) Micheli (syn. *Aquarius grandiflorus* (Cham. & Schltdl.) Christenh. & Byng, *Alisma grandiflorum* Cham. & Schltdl.) (Alismataceae) are plant species popularly known as *chapéu-de-couro* in Brazil. The aerial parts of these species are traditionally used to treat rheumatism and arthritis in the country, among other uses [1,2]. Both species occur in tropical regions of South America [3], being predominantly found in the northeast and central-west regions of Brazil, mainly in floodplains, swamps, and coastal areas [4].

The first and second editions of the *Brazilian Pharmacopeia* contain a monograph of *E. macrophyllus* [5,6], while a monograph of *E. grandiflorus* is found in the fifth and sixth editions [7,8]. These two species are utilized for similar medicinal purposes in various regions of Brazil as teas or herbal preparations [2]. The interchangeable use of *E. macrophyllus* and *E. grandiflorus* may be attributed to difficulties in distinguishing them based on morphological characteristics. Both species are aquatic plants with large leathery leaves, which have led to their popular name *chapéu-de-couro* (leather hat) [9]. *E. macrophyllus* and *E. grandiflorus* share inflorescences with delicate white flowers, which are also used for decorative purposes, and their leaf and floral anatomy do not exhibit noticeable differences to non-specialists. The presence of phenotypic plasticity and the striking similarity in morphological structures [10] makes it challenging to differentiate these species unequivocally.

The chemical composition of leaves from *E. macrophyllus* and *E. grandiflorus* has been extensively investigated, revealing similarities that include the presence of diterpenes, flavone C-glycosides, hydroxycinnamoyl tartaric acid derivatives, alkaloids, saponins, and phenolic acids [11,12,13,14]. Moreover, the observed biological activities of these species have been linked to their chemical composition. For instance, a flavonoid-rich fraction from *E. macrophyllus* exhibited a more potent anti-inflammatory effect than the extract in the air pouch model in mice, reducing leukotriene B_4_ release and neutrophil migration in vitro [11]. The anti-arthritic activity of *E. grandiflorus* leaves has been attributed to flavone C-glycosides, as evidenced by the potent effect observed when administering a fraction enriched in these compounds in an antigen-induced arthritis model in mice [13]. Additionally, the flavonoid-rich fraction elicited the most potent inhibition of tumor necrosis factor (TNF) release by lipopolysaccharide (LPS)-stimulated THP-1 cells, along with its constituents isovitexin and isoorientin [12]. Other classes of metabolites present in these species, such as diterpenes, hydroxycinnamoyl tartaric acid derivatives, and *cis*- and *trans*-aconitic acid, have also been reported for their anti-inflammatory activity by inhibiting TNF release in LPS-stimulated THP-1 cells [12].

Although the leaves of *E. macrophyllus* and *E. grandiflorus* are consumed locally for medicinal purposes, there are no clear-cut differences based on morphological characteristics or chemical composition that can be used to unambiguously differentiate them. Therefore, the aim of this study was to undertake molecular, chemical, and biological investigations of different extracts prepared from *E. macrophyllus* and *E. grandiflorus* leaves in order to highlight similarities and differences between these species. The extracts were analyzed by UPLC-DAD (ultra-efficiency chromatography coupled to diode array detector) for the quantification of chemical markers (*cis*- and *trans*-aconitic acid, homoorientin, chicoric acid, swertisin, caffeoyl-feruloyl-tartaric acid, and di-feruloyl-tartaric acid), using a method developed and validated in the present work. The extracts had their anti-oxidant activity assayed in vitro, along with their effect on the release of TNF by LPS-stimulated THP-1 cells, to access their potential anti-inflammatory effect. Finally, a multi-variate exploratory principal component analysis (PCA) model [15,16] was built, aiming to investigate the possible existence of correlations between the quantitative chemical composition of the extracts and their biological effects.

## 2. Material and Methods

### 2.1. Plant Drug Samples

The leaves of *E. macrophyllus* and *E. grandiflorus* were obtained from Indústria Farmacêutica Catedral (Belo Horizonte, Brazil). Two lots of the first species were purchased and labeled as DV1 (April 2014) and DV2 (March 2019), along with a lot of the second species identified as EG (November 2019). These plant drugs were provided dried and underwent quality control checks as indicated in the supplier’s reports. Upon receipt, the leaves were pulverized using a knife mill (Marconi, São Paulo, Brazil) and stored in glass flasks for further use. A third sample of *E. macrophyllus* aerial parts was collected at the Natural History Museum of the Universidade Federal de Minas Gerais (UFMG) and a voucher was incorporated into the BHCB herbarium at UFMG, under the number BHCB 28557. After drying in a ventilated oven at 40 °C, this plant (drug-coded DVMus) was pulverized in a knife mill and stored in a glass flask.

### 2.2. Chemicals and Reagents

All chemicals, reagents, and reference compounds used were of analytical or LC-MS (liquid chromatography coupled to mass spectrometer)/UPLC grade. The water was filtered through a Milli-Q water purification system (Millipore, Bedford, MA, USA) before use. *trans*-aconitic acid (purity > 99.0%), chicoric acid (purity > 98%), and homoorientin (purity > 99%) were purchased from Extrasynthese, France; *cis*-aconitic acid (purity > 98%), 1,1-diphenyl-2-picryl-hydrazyl-hydrate (DPPH), pyrogallol (purity > 98%), quercetin (purity > 95%), β-carotene (purity > 93%), lipopolysaccharide from *Escherichia coli* (Sigma-Aldrich, St. Louis, MO, USA), *o*-phenylenediamine (OPD), dexamethasone (purity > 98%), and 3-(4,5-dimethyl-thiazol-2-yl)-2,5-diphenyltetrazolium bromide were obtained from Sigma-Aldrich (St. Louis, MO, USA). Swertisin (purity > 95%) was kindly donated by Prof. Eloir Paulo Schenkel (Universidade Federal de Santa Catarina, Brazil). Human TNF-α DY210 Duo Set was acquired from R&D Systems (Minneapolis, MN, USA).

### 2.3. DNA Isolation, Amplification, Sequencing, Alignment, and Pairwise Distance Calculation

Total genomic DNA was extracted from fresh leaves using a modified version of the 2 × CTAB protocol of Ref. [17]. For PCR amplifications, we used primers described in the Supplementary Table S1 and the parameters previously described [10,18], with minor modifications. The amplified and purified products were sequenced on both DNA strands using the same PCR primers by Macrogen Inc. (Seoul, Korea). The DNA sequence electropherograms were edited and a consensus generated through the Staden program [19]. The sequences were aligned using the MUSCLE program [20] and thereafter manually adjusted using the MEGA7 program [21], according to the procedures described by Ref. [22]. Pairwise distances of the combined dataset (47 terminals and 3182 base pairs) between the major groups recovered in the phylogenetic analyses were calculated using the *p*-distance method in MEGA7 [21]. Gaps and missing data were treated with the pairwise deletion option.

### 2.4. Molecular Markers, Taxon Sampling, and Molecular Phylogenetic Analyses

Nucleotide sequences from three nuclear genome regions, the nrITS consisting of ITS1, ITS2 and the intervening 5.8S gene, the 5S non-transcribed region (5S-NTS) and the second intron of *LEAFY*, and two plastid regions (*matK-trnK* and *psbA-trnH*) were used in the analysis. Marker selection was based on previous molecular phylogenetic analyses of *Echinodorus* [10,18]. The taxon sampling was based on a selection of taxa available in GenBank selected to cover the taxonomic diversity of *Echinodorus* and included 38 taxa and 47 terminals. Voucher information, geographic origins, and GenBank accession numbers are provided in Supplementary Table S2. To root the trees, we used *Echinodorus berteroi*, which was recovered as sister to the other species of the genus according to the results of Lehtonen and [10,18]. Most species of *Echinodorus*, except *E. berteroi* (the type species of the genus), have recently been transferred to the genus *Aquarius* Christenh. & Byng [23]. An evaluation of this proposal was beyond the objectives of this study and we followed the previous classification here, keeping all species in *Echinodorus*.

To compare the outcomes of different methodologies in phylogenetic analysis, we employed both maximum parsimony (MP) and Bayesian inference (BI) techniques. Our investigation focused on evaluating a model-free nucleotide substitution approach (MP) in comparison with a model-based approach (BI). We conducted an extensive examination of individual markers and a merged dataset to detect any potential inconsistencies. For the maximum parsimony analysis, we utilized PAUP* version 4 [24] and implemented Fitch parsimony with equal weights for unordered characters (Fitch, 1971) as the criterion for determining optimality. Each search involved 2000 replicates, with random taxon additions; branch swapping was carried out using the tree-bisection and reconnection (TBR) algorithm. To mitigate excessive swapping on suboptimal islands, we stored a maximum of 10 trees per replicate. To evaluate the internal support, we employed character bootstrapping [25], with 2000 replicates using simple addition, and TBR branch swapping. Throughout the bootstrapping process, we retained up to 15 trees per replicate. The assessment of bootstrap support levels relied on bootstrap percentages (BP), where values falling between 50% and 70% were classified as weak, 71% and 85% as moderate, and values exceeding 85% as strong [26].

Bayesian analysis using MrBayes 3.2.7a [27] was implemented in the Cyberinfrastructure for Phylogenetic Research (CIPRES) Portal 2.0 (San Diego Supercomputer Center, La Jolla, CA, USA [28], treating each DNA region as a separate partition. An evolutionary model for each DNA region was selected in MrModeltest 2 [29] using the hierarchical likelihood ratio tests (hLRTs). To estimate model parameters for each partition separately, we utilized the unlink command. Our analysis involved conducting two independent runs, each with four chains, spanning a total of 10,000,000 generations. During the Markov chain Monte Carlo (MCMC) sampling, we sampled one tree every 1000 generations, employing a temperature parameter of 0.2. We assessed convergence between the runs using multiple criteria. The average standard deviation of split frequencies was evaluated and confirmed to be less than 0.01, indicating convergence. Additionally, we calculated the potential scale reduction factor (PSRF), which yielded a value of 1.0, further validating convergence. Convergence was achieved after 1,970,000 generations. We discarded the initial 2500 trees (25%) as burn-in and utilized the remaining trees to evaluate the topology and to calculate posterior probabilities (PP) through a 50% majority-rule consensus. It is worth noting that, in Bayesian analysis, posterior probabilities cannot be directly compared with bootstrap percentages (BP), as PP values tend to be higher [30]. To evaluate support, we applied criteria similar to a standard statistical test. Groups with a PP value exceeding 0.95 were considered strongly supported, while PP values ranging from 0.90 to 0.95 were categorized as moderately supported. Groups with PP values below 0.90 were considered weakly supported.

### 2.5. Preparation of Extracts

Portions of 1.0 g of the dried leaves of *E. macrophyllus* (DV1, DV2, DVMus) and *E. grandiflorus* were sonicated with 150 mL of 96 °GL EtOH or with hydroethanolic solutions at 90% (90% EtOH *v*/*v*), 70% (70% EtOH *v*/*v*), and 50% (50% EtOH *v*/*v*) in 3 cycles of 10 min each. The ethanol content of the hydroethanolic solutions was adjusted with the aid of an alcoholmeter. The obtained extracts were filtered, pooled, and concentrated in a rotary evaporator, under reduced pressure, at an average temperature of 55 °C. Subsequently, the hydroethanolic extracts were lyophilized for complete elimination of residual water. The extracts were transferred to glass flasks and kept in a desiccator until constant weight for yield calculation.

### 2.6. Quantification of Chemical Markers by UPLC-DAD

The contents of the chemical markers *cis*- and *trans*-aconitic acid, homoorientin, chicoric acid, swertisin, caffeoyl-feruloyl-tartaric acid, and di-feruloyl-tartaric acid were quantified in the extracts described in Section 2.5, using an UPLC-DAD method developed and validated by us.

#### 2.6.1. Sample Preparation and Chromatographic and Analytical Conditions

For the analysis, portions (2.00 mg) of the samples were dissolved with 1.0 mL of MeOH/H_2_O (1:1 *v*/*v*) for 70% EtOH and 50% EtOH extracts or solubilized in methanol for 96 °GL EtOH and 90% EtOH extracts. The extracts were sonicated in an ultrasound bath for 10 min, at room temperature. Thereafter, the extracts were centrifuged at 8400× *g* for 10 min and filtered through a 0.22 μm membrane of PTFE; the obtained supernatants (5 µL) were injected onto the UPLC system.

A *Waters Acquity UPLC* H-Class Bio System (2015 *model*) system composed of a quaternary pump, an autosampler, a photodiode array detector (DAD) 2996, and a Waters Empower pro data handling system was employed (Waters Corporation, Milford, MA, USA). The analyses were performed on a BEH Shield RP-18 column (100 × 2.1 mm i.d., 1.7 µm; Waters) in combination with a BEH Shield RP-18 guard column (4 × 4 mm i.d., 1.7 µm; Waters) at 40 °C. Water (A) and acetonitrile (B) were used as eluents, both containing 0.1% (*v*/*v*) of formic acid, at a flow rate of 0.3 mL/min. A segmented gradient was employed for elution, comprising concave (type 7) and linear gradient steps, along with isocratic elution steps, as follows: 0 min, 4% B; 1.5 min, 15% B (type 7 gradient); 2.5 min, 16.2% B (linear gradient); 3.5 min, 16.2% B; 4.0 min, 17.4% B (type 7 gradient); 5.0 min, 17.4% B (isocratic); 8.5 min, 20.6% B (type 7 gradient); 9.5 min, 20.6% B (isocratic); 10.5 min, 21.3% B (linear gradient); 11.5 min, 21.3% B (isocratic); 30.5 min, 90% B (type 7 gradient); 32.5 min, 95% B (linear gradient); 33.5 min, 95% B (isocratic). A reverse gradient was adopted to return to the initial elution conditions (4% B) in 1 min; a 5-min equilibrium time was adopted between injections. UV spectra from 190 to 400 nm were recorded online; chromatograms were extracted at 350 nm to quantify flavonoids and tartaric acid derivatives and at 220 nm for aconitic acids quantitation.

#### 2.6.2. Identification of Chemical Markers by LC-MS Analysis

The identity of the chemical markers found in the extracts was confirmed by ultra-performance liquid chromatography with electrospray ionization and *triple quadrupole* mass spectrometry (UPLC-ESI-MS) analysis, carried out in a Waters *Acquity* UPLC system (Waters, Milford, MA, USA), composed of a binary pump, an auto sampler, an in-line degasser, a photodiode array detector (Waters), and a mass spectrometer Xeco™ Triple Quadrupole MS (Waters). Data were processed using MassLynx4.1 (Waters). The chromatographic conditions were those described for the UPLC-DAD method (Section 2.6.1). The mass spectrometer was operated in the exploratory mode (scan), both at the negative and positive ionization modes using the following conditions: capillary voltage of 3.54 kV; 10–70 V ramp cone voltage; source temperature 120 °C; desolvation temperature 450 °C. Mass/charge ratios (*m*/*z*) from 100 to 900 were evaluated. The samples were injected at 1 mg/mL, obtained by solubilizing 1 mg of each extract in 1.0 mL of methanol HPLC grade. Extracts were sonicated in an ultrasound bath for 10 min and centrifuged at 8400× *g* for 10 min; the supernatants were filtered through a PVDF membrane (0.22 µm) and automatically injected into the UPLC-ESI-MS system.

#### 2.6.3. Validation of the UPLC-DAD Method

The method was validated according to the guidelines of Refs. [31,32,33]. The 70% EtOH extract was selected for validation studies because of its higher complexity compared with other extracts.

Selectivity and system suitability. Purity of peaks from the analytes was evaluated by UV spectra recorded by DAD at different points, in chromatograms of sample solutions, and analyzed by the relationship between the purity angle and the threshold. System suitability was assessed by the outcomes for resolution, retention factor, tailing, number of theoretical plates, and RSD (relative standard deviation) of the retention time of peaks, calculated by Empower 3 software (Waters, Milford, MA, USA).

Linearity, precision, limit of quantification (LOQ), and limit of detection (LOD). Standard solutions were prepared for each compound using a 1:1 mixture of *cis*- and *trans*-aconitic acid (6.25, 12.5, 25.0, 50.0, and 100.0 µg/mL), homoorientin (1.25, 2.5, 5.0, 10.0, and 20.0 µg/mL), chicoric acid (9.37, 18.75, 37.5, 75.0, 150.0, and 300 µg/mL), and swertisin (30.0, 45.0, 67.0, 100.0, and 150.0 µg/mL). The solvents used for preparing the standard solutions were either MeOH or ultrapure water, depending on the solubility of each substance. Triplicate aliquots (5 µL) of these prepared solutions were injected onto the UPLC-DAD system using the developed method. The calibration curves were constructed by analyzing the injected mass of each compound and quantifying the corresponding peak areas. The analysis was performed on two independent days and linear regression analysis was employed to determine the calibration curves using Prism 6.01 software (GraphPad, San Diego, CA, USA). The calibration curve of chicoric acid was also used to quantify the contents of caffeoyl-feruloyl-tartaric acid and di-feruloyl-tartaric acid. The intra-day precision was evaluated by calculating the relative standard deviation (RSD) values of sample solutions analyzed on the same day (*n* = 6) at 100% of the analyte concentration. Similarly, the inter-day precision was assessed by analyzing the sample solutions on two different days, performed by two different analysts (*n* = 12). The limits of quantification (LOQ) and detection (LOD) were determined by injecting standard solutions at progressively lower concentrations, in quintuplicate. The LOQ was determined as the concentration at which the peak area exhibited an RSD below 3%, while the LOD was defined as the concentration that yielded a signal-to-noise ratio (S/N) of 3.

Recovery and robustness. Accuracy of the method was accessed by recovery studies.

Recovery was assessed by adding known aliquots of reference compound solutions to the extracts at three concentration levels falling within the calibration curve. To evaluate robustness, six sample solutions were prepared and analyzed using both the established conditions and by altering the following parameters: column temperature (38 and 42 °C), wavelength (352 and 348 nm to flavonoids and phenolic acids; 218 and 222 nm to aconitic acids), and formic acid content in the mobile phase (0.08 and 0.12% *v*/*v*). The concentrations of the analytes were compared by ANOVA, followed by Tukey’s test (*p* < 0.05).

### 2.7. Effect on the Release of TNF In Vitro

The effect of the extracts on the release of TNF was evaluated in vitro in THP-1 cell culture (monocyte lineage derived from human acute monocytic leukemia: ATCC TIB-202) stimulated by LPS. Cell viability was evaluated by the MTT (3-4,5-dimethyl-thiazol-2-yl-2,5-diphenyltetrazolium bromide) assay, as previously described, with modifications [34]. A cell suspension was prepared at 1.0 × 10^6^ cells/mL, obtained by centrifuging a culture flask and sequentially resuspending the pellet with RPMI 10% FBS and 2 µL PMA (1 mg/mL). Then, 100 µL aliquots of this suspension were transferred to the wells of a microplate, which were incubated at 37 °C in a 5% CO_2_ atmosphere for 6 h to allow cell differentiation and adhesion. After this period, the cells were starved with RPMI medium, supplemented with 2% FBS, and incubated again under the same conditions for 12 h. The cells were transferred to a 96-well plate at 1 ×10^6^ cells/well and incubated for 24 h in an oven at 37 °C and a humidified atmosphere of 5% CO_2_. Subsequently, 100 μL of complete medium with 2% of FBS containing different concentrations of the extracts (60 µg/mL) were added in triplicate and the plate was incubated for 3 h under the conditions described above. Then, 20 µL of LPS (1 ng/mL) were added to the wells and the plate was incubated again for 24 h under the same conditions. After incubation, the microplate was centrifuged (1800× *g* for 5 min) and 100 µL of the supernatant was collected to quantify cytokines by the ELISA method, according to the protocol described in the sequence. The cell pellet was used to assess cell viability by MTT and 28 μL/well of tetrazolium salt (MTT) was added at a concentration of 2 mg/mL in phosphate buffer (PBS). After 90 min of incubation with the MTT, the entire contents were aspirated by a Pasteur pipette, followed by the addition of DMSO to each well (100 μL/well) to solubilize the formed formazan crystals. The microplates were read in a spectrophotometer at 510 nm. Cell viability was calculated according to the equation (A − B/C − B) × 100, where A, B, and C are the absorbance of the samples, blank, and negative control, respectively.

#### ELISA Protocols for In Vitro Assays

The assay was conducted using an ELISA kit from R&D Systems (DY210-KIT TNF), following the manufacturer’s instructions and guidelines. An amount of 100 µL/well of TNF capture antibody (4 µg/mL), solubilized in PBS, was added to a 96-well microplate and the plate was kept at 4 °C for 12 h. After incubation, the plate was washed (PBS/Tween 20, 0.1% *v*/*v*) and completely dried. Thereafter, 200 µL of the blocking solution (1% bovine serum albumin in PBS) was added. After 2 h, the plate was washed and dried as described above. Then, 100 µL of samples or TNF standard solution (1000, 500, 250, 125, 62.5, 31.25, and 15.6, pg/mL) was added. The microplate was incubated for 18 h at 4 °C and then the plates were washed and dried. In the sequence, 100 µL of TNF detection antibody solution (500 ng/mL) was added. After 2 h, the plates were washed and dried and then 100 µL of a solution containing streptavidin bound to peroxidase (R&D system) was added to the microplate wells. The plate was incubated for 30 min and washed and dried as described above. Subsequently, 100 µL of *o*-phenylenediamine (OPD) in citrate buffer (0.4 mg/mL) and 2.4 µL of hydrogen peroxide solution (35% *v*/*v*) were added to the wells. The reaction was stopped after 30 min by adding 50 µL of 1 mol/L hydrochloric acid. The OPD oxidation product was detected in a microplate reader at 490 nm. The concentration of TNF was calculated using the calibration curve.

### 2.8. In Vitro Anti-Oxidant Activity

#### 2.8.1. DPPH Radical Scavenger Activity

The assessment of anti-oxidant activity by the 2,2-diphenyl-1-picrylhydrazyl radical (DPPH) assay was carried out as previously described [35], with adaptations. All extracts were initially tested at 200 μg/mL; subsequently, the active samples were evaluated at 1–200 μg/mL and concentration–response curves were constructed. To carry out the experiments, 250 μL of each sample, methanol (negative control), and pyrogallol (50 μg/mL, positive control) were added to a 96-well plate. Then, 100 μL of methanol (blank wells) and DPPH (120 μg/mL, reaction wells) were added to the microplate. The measurements were carried out using a microplate reader (EL808IU-Biotek, Winooski, VT, USA) at 515 nm, with readings taken every 5 min for a total of 25 min. The percentage of radical scavenging activity (% RSA) was determined using the following formula: % RSA = (AC − AS)/AC) × 100, where AC represents the absorbance of the control and AS represents the absorbance of the samples taken at 35 min. The results were expressed as the EC_50_ value, which was the effective concentration at which 50% of DPPH radicals were scavenged. The EC_50_ was determined through non-linear regression analysis using GraphPad Prism, version 6.0.

#### 2.8.2. β-Carotene/Linoleic Acid Co-Oxidation Assay

The assays were carried out as previously described [36], with minor adaptations. The extracts were solubilized in methanol (2.2 mg/mL) and subsequently diluted from 2.47 to 200 μg/mL (dilution factor 1:3). For the experiments, 25 μL aliquots of the solutions were added to a 96-well plate. A mixture of 25 mg of linoleic acid and 100 mg Tween 20 (Sigma, St. Louis, MO, USA) was added to a round-bottom flask containing 1 mL of a β-carotene solution in chloroform (1 mg/mL, Sigma) and transferred to a rotary evaporator for complete evaporation of the solvent. Then, 50 mL of aerated water was added to the flask, affording the β-carotene emulsion. A blank emulsion was prepared similarly, except for the addition of the β-carotene solution. Aliquots of 250 μL from both emulsions were added to the even and odd columns of the microplate. Methanol was used as a negative control and quercetin (20 µg/mL, Sigma) was used as a positive control. Readings were taken immediately at a wavelength of 470 nm and subsequently at 15 min intervals up to 120 min. Throughout the reading period, the microplate was maintained at an incubation temperature of 45 °C. The anti-oxidant activity was expressed as % of inhibition of lipid peroxidation (%I), using the equation I% = control absorbance (Ac) (initial abs − final abs) − sample absorbance drops (Aam) (final abs − initial abs)/Ac × 100). Assays were performed in triplicate and IC_50_ values were determined by non-linear regression using GraphPad Prism, version 6.0 (GraphPad Software, San Diego, CA, USA).

#### 2.8.3. ROS Activity in THP-1 Cells

The effect of the extracts on the production of reactive oxygen species (ROS) by THP-1 cells was performed according to [37] with adaptations. THP-1 cells were cultured in RPMI medium supplemented with 10% fetal bovine serum (FBS). A cell suspension was prepared at a density of 6 × 10^5^ cells/mL, obtained by centrifuging a culture flask and sequentially resuspending the pellet with RPMI 10% FBS and 4 µL PMA (100 nM), following incubation for 72 h in an oven at 37 °C and 5% CO_2_. Thereafter, PMA was washed with RPMI 10% FBS, with subsequent incubation of the cells without adding PMA and 2% of FBS in an oven at 37 °C and 5% CO_2_ for 24 h. Subsequently, 1 × 10^6^ cells per well were placed (250 μL) in a 24-well plate and incubated for 24 h in an oven at 37 °C and 5% CO_2_. After incubation, the cells were treated with the extracts (60 μg/mL in RPMI with 0.2% DMSO) and the plate was incubated for another 3 h in the previous conditions. Then, the cells were treated with 20 µL of a LPS solution (10 μg/mL) at 15 min intervals up to 75 min. The following controls were added to the plate: wells containing only RPMI medium without LPS, wells containing RPMI medium with LPS, and wells containing non-differentiated THP-1 cells. Quercetin (100 μmol/L in methanol) was used as a positive control. Following LPS stimulation, the cells were incubated with 10 μM H_2_DCFDA (2′,7′-dichlorodihydrofluorescein diacetate) for 15 min at 37 °C and 5% CO_2_. The supernatants were collected and fluorescence was read in a microplate reader (Varioskan™ LUX, Thermo Scientific, Waltham, MA, USA) at 493 nm for excitation and at 522 nm for emission.

### 2.9. Statistical Analysis

The concentrations of the analytes were compared using analysis of variance (ANOVA), followed by Tukey’s test for post hoc multiple comparisons, with a significance level set at *p* < 0.05. Statistical analysis was performed using GraphPad Prism software, version 6.0 (GraphPad Software Inc.). The results were expressed as mean ± standard deviation (SD) and differences were considered statistically significant when *p* < 0.05.

### 2.10. PCA Model

Principal component analysis (PCA) was performed employing MATLAB R2010b software (The MathWorks, Natick, MA, USA) jointly with PLS_Toolbox 6.5 (Eigenvector Technologies, Manson, IA, USA).

## 3. Results and Discussion

### 3.1. Molecular and Phylogenetic Analysis of E. macrophyllus

The BOLD (Barcode of Life Data System) database of *Echinodorus* is completely based on GenBank sequences and therefore we used BLASTN (nucleotide blast) on the NCBI BLAST homepage [38] to search in the nucleotide collection database and to identify the sequences/taxa with higher identity with the sequences we generated from *E. macrophyllus*. The best match varied between markers (Table 1). For *psbA-trnH*, *matK-trnK,* and Leafy, the best match was with *E. paniculatus*, while, for ITS, the best match was with *E. osiris*, but the differences between the additional hits were very small.

**Figure 1:**
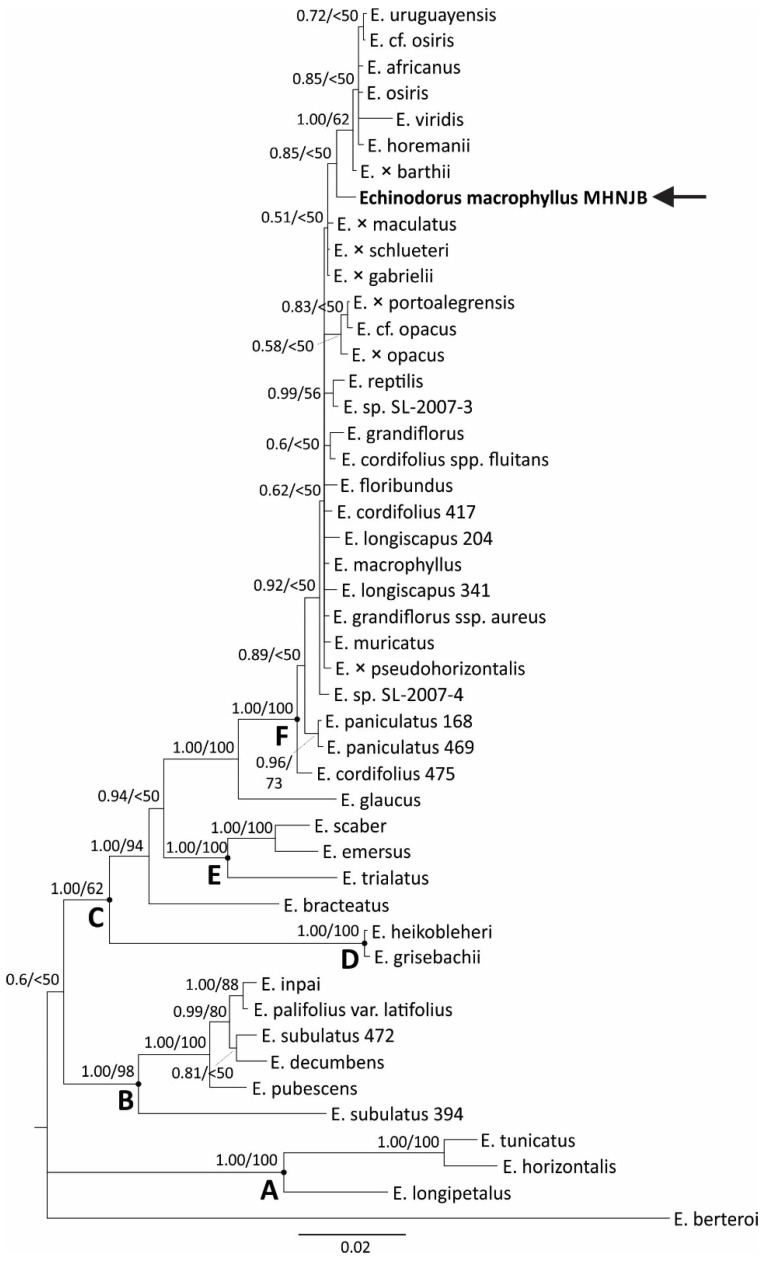
Bayesian 50% majority-rule consensus tree of the combined ITS, 5S-NTS, LEAFY, *matK-trnK*, and *psbA-trnH* datasets. Numbers above the lines, next to the nodes, represent the posterior probabilities (PP) from the Bayesian analysis and bootstrap percentages (BP) from parsimony analysis. Major clades discussed in the text are indicated by the letters A to F. The sequenced sample from *E. macrophyllus* is highlighted in bold and by an arrow. The generic names for all *Echinodorus* species are abbreviated.

Since an unequivocal match was not possible, we performed phylogenetic analysis to verify which taxa were most related to the sequenced *E. macrophyllus* sample using the ITS, 5S-NTS, Leafy, *matK-trnK*, and *psbA-trnH* markers. Initial analysis with each marker individually did not detect any case of strongly supported incongruence, so a combined matrix was used for the final analysis. Table 2 presents the general features of the datasets and parsimony statistics, along with a summary of the models implemented for each partition. The strict consensus tree from the parsimony analysis and the Bayesian majority-rule consensus tree were for the most part congruent and, as the latter was more fully resolved and had stronger overall support, it was chosen for presentation and discussion (Figure 1). Our analysis recovered three main clades with high support within *Echinodorus*: clade A (PP 1.00, BP 100%), clade B (PP 1.00, BP 98%), and clade C (PP 1.00, BP 62%) (Figure 1). Clade C in turn was divided into three main clades: a clade formed by *E. grisebachii* and *E. heikobleheri* (clade D: PP 1.00, BP 100%), another formed by *E. trialatus*, *E. emersus,* and *E. scaber* (clade E: PP 1.00, BP 100%), and clade F (PP 1.00, BP 100%), which included our sample of *E. macrophyllus* and 20 other taxa. Relationships within clade F were poorly resolved and poorly supported. Our sample of *E. macrophyllus* was recovered as sister to a clade containing *E. osiris* and six other taxa, but with low support (PP 0.85, BP < 50%). By using neither analysis, our *E. macrophyllus* sample was recovered as closest to the *E. macrophyllus* sample available in GenBank. This result was probably due to a combination of factors. First, sequence divergence between *Echinodorus* species was very low. Mean pairwise distances within and between the major clades identified in the molecular phylogenetic analysis (Figure 1) showed low values, particularly within the clades (Table 3). In clade F, for example, which included the *E. macrophyllus* samples, the mean pairwise distance among the sequences of all markers was only 0.007; for some markers, such as *matK-trnK*, these values were even lower (Table 4). These results suggested a recent origin and diversification of some species groups of the genus and indicated that the combination *matK* and *rbcL*, for example, (proposed as the standard for plant DNA barcoding [39]) in most cases would not be sufficient for the discrimination of closely related species of *Echinodorus*. Second, the sampling of *Echinodorus* in nucleotide collection databases was still small relative to the breadth of geographic distribution and morphological variability of the species. For *E. macrophyllus*, for example, distributed throughout Brazil and also in Bolivia [40], there was only one sample available in GenBank. Finally, it was also not possible to rule out the possibility of conflicting identifications between our sample and those of the GenBank.

In general, the identification of related and morphologically similar species of *Echinodorus* is difficult and requires specialized taxonomic expertise. The use of DNA sequences can represent a great differential in this sense, particularly when it comes from the certification of dried samples processed for the industry, when the identification based on morphological characters is not possible. However, this will require increased population and geographic sampling of species and the use of more polymorphic and more informative markers, such as the nuclear markers 5S-NTS and Leafy, which proved to be the most diversified among those tested (Table 4) and thus may be good candidates for an initial examination.

### 3.2. Development and Validation of an UPLC-DAD Method for the Analysis of Chemical Markers

Aiming to investigate the chemical composition of *E. macrophyllus* and *E. grandiflorus*, different extracts were prepared by sonicating the dried leaves with distinct solvents. The extracts prepared with 50% EtOH and 96 °GL EtOH showed the highest and lowest yields, respectively (Table 5). These findings indicate the predominance of hydrophilic compounds in both species. UPLC-DAD profiles were initially recorded for all extracts using an exploratory run and pointed out the 70% EtOH extract as the one with the most complex matrix. Therefore, this extract was employed for development and validation of the analytical method to quantify chemical markers.

**Figure 2:**
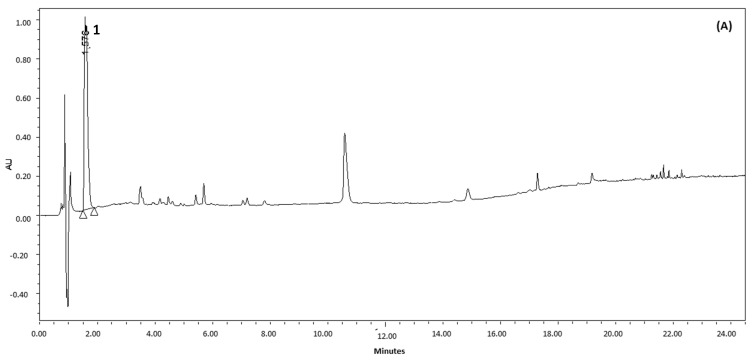

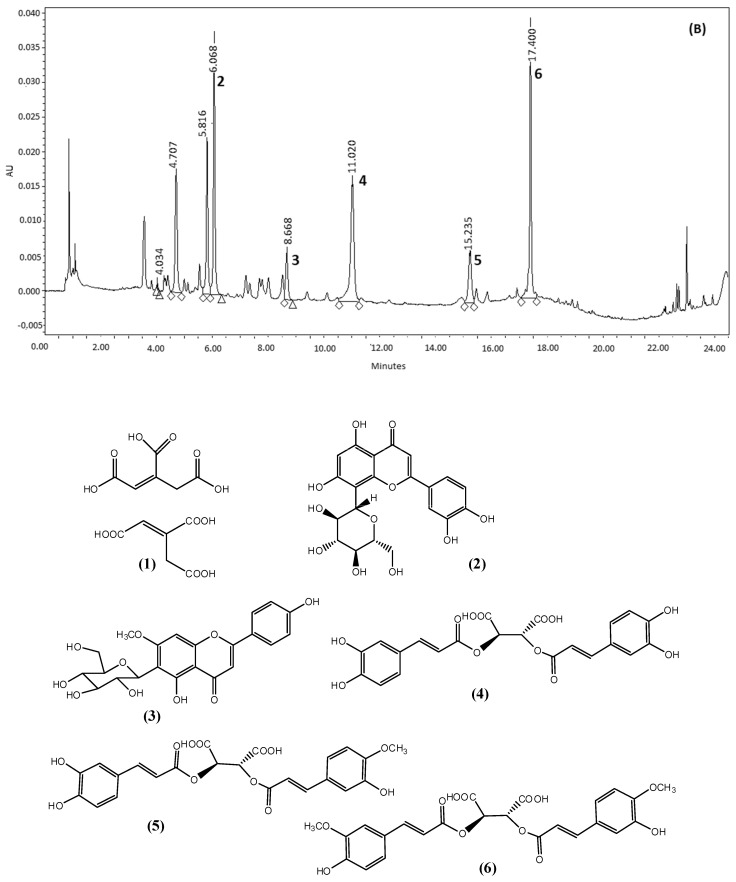
Typical UPLC-DAD chromatograms obtained for 70% EtOH extract of *E. macrophyllus* leaves. The chemical structures and corresponding peaks are (**1**) *cis*- and *trans*-aconitic acid, (**2**) homoorientin, (**3**) swertisin, (**4**) chicoric acid, (**5**) caffeoyl-feruloyl-tartaric acid, and (**6**) di-feruloyl-tartaric acid. Chromatograms registered at 220 nm (**A**) and 350 nm (**B**). Chromatographic conditions: see Materials and Methods (Section 2.6.1).

A segmented gradient (concave and linear) of water and acetonitrile, intercalated with isocratic elution steps, was used for UPLC analysis. The elution conditions were set and optimized to allow the adequate separation of compounds **1**–**6** in extracts of leaves from *E. grandiflorus* and *E. macrophyllus.* Two different wavelengths were used for quantifying the analytes: at 220 nm for the analysis of *cis*- and *trans*-aconitic acid (Figure 2A) and at 350 nm to quantify flavonoids and tartaric acid (Figure 2B).

The identification of the chemical markers in the extracts was performed by the analysis of reference compounds (*cis*- and *trans*-aconitic acid, homoorientin, chicoric acid, and swertisin) in the established chromatographic conditions and comparison of their retention times and UV data recorded by DAD with those of peaks registered in the chromatograms. The identity of the chemical markers was further confirmed by UPLC-ESI-MS analysis. Two other constituents of the extracts were assigned by UPLC-ESI-MS analysis: caffeoyl-feruloyl-tartaric acid (**5**) and di-feruloyl-tartaric acid (**6**). Compound **5** produced the deprotonated anion [M-H]^−^ at *m*/*z* 487 Da in the negative ionization mode, along with fragment ions at *m*/*z* 149 Da [M-338]^−^, ascribed to tartaric acid, at *m*/*z* 193 Da [M-294]^−^, credited to the feruloyl unit, and at *m*/*z* 311 Da [M-176]^−^, resulting from the loss of the caffeoyl unit. The identification of this compound by LC-MS analysis in a fraction from the ethanolic extract of *E. macrophyllus* was reported by [11]. In its turn, compound **6** produced the molecular ions at *m*/*z* 501 [M-H]^−^ in the negative ionization mode, which, along with the fragment ions observed at *m*/*z* 149 [M-352]^−^, was ascribed to tartaric acid, and at *m*/*z* 325 [M-176]^−^, credited to the loss of one of the caffeoyl units, thus allowing its assignation. To the best of our knowledge, compounds **5** and **6** have not been isolated so far from *E. macrophyllus*. On the other hand, several caffeoyl-feruloyl-tartaric acid derivatives along with cinnamoyl-tartaric acid derivatives were obtained from *E. grandiflorus* [41].

Among the compounds identified in the extracts of *E. macrophyllus*, only homoorientin [42], swertisin [43], and caffeoyl-feruloyl-tartaric acid [11] have been previously reported; the occurrence of all other constituents is herein described for the first time. On the other hand, all of them have been previously reported as constituents of *E. grandiflorus* [12,13,41], corroborating the similar chemical composition of these species.

In the sequence, the developed UPLC-DAD method was validated for the quantitation of the six chemical markers identified in the extracts (Figure 2). The selectivity was assessed by spectral homogeneity of the peaks, evaluated by DAD. The ascending, upper, and descending regions of the spectrum of each peak matched exactly, indicating that other compounds were not co-eluted, i.e., the threshold angle was higher than the purity angle. The reliability of the chromatographic conditions was assured by the results of system suitability tests, as depicted in Table 5. Relative standard deviation (RSD) values of retention time (≤1%), number of theoretical plates (N > 2000), retention factor (*k* > 0.5), USP tailing factor (≤2.0), and resolution (Rs > 1.5) obtained for all analytes fell within the limits recommended by [31,44] for quantitative analysis.

The calibration curves constructed for the reference compounds were linear along the established ranges, with *r*^2^ values above 0.99 (Table 6). Curves obtained in two consecutive days were statistically similar (*p* > 0.05). Furthermore, statistical analysis showed that calibration data presented normal distribution, with randomly distributed residuals. The method showed good precision with RSD values below 4.3% for all analytes (Table 6). Additionally, the mean concentrations determined for the inter-day precision experiment did not differ statistically (*p* > 0.05) for all analytes. LOQ values between 0.04 and 1.92 μg and LOD between 0.01 and 0.58 μg (Table 6) demonstrated the high sensitivity of the method. The method demonstrated robustness (*p* > 0.05) under all tested conditions, as the introduced variations did not significantly alter the concentrations of the analytes when compared with the results obtained using the established conditions. The method was also accurate, as indicated by the recovery rates ranging from 95.8% to 110.8% for all tested compounds. The results of the method validation are summarized in Table 6.

### 3.3. Quantitation of Chemical Markers in Extracts of E. macrophyllus and E. grandiflorus

The developed UPLC-DAD method was applied to the analysis of 12 extracts (96 °GL EtOH, 90% EtOH, 70% EtOH, and 50% EtOH) prepared from leaves of three different samples of *E. macrophyllus* (commercial lots DV1 and DV2; cultivated sample DVMus), along with four extracts of *E. grandiflorus* (96 °GL EtOH, 90% EtOH, 70% EtOH, and 50% EtOH) prepared from a single commercial lot. The quantitative results expressed as percentage mean (*w*/*w*) ± SD of triplicates are depicted in Table 7. As expected, all extracts prepared with 50% EtOH and 70% EtOH showed high concentrations of the polar compounds *cis*- and *trans*-aconitic acid, analyzed jointly **(1)**, with variable contents in the samples. The 70% EtOH extracts obtained from DV1 (8.09 ± 0.02% *w*/*w*) and DVMus (7.47 ± 0.03%) showed similar contents of **1**, whereas the highest concentration was measured in the 50% EtOH of *E. grandiflorus* (20.12 ± 0.16%) (Table 7).

Homoorientin (**2**) was found in all extracts prepared with 70% EtOH and 90% EtOH, with variable contents. The extracts obtained from DVMus (70% EtOH, 0.02 ± 0.00%; 90% EtOH, 0.04 ± 0.01%) presented the lowest concentration of **2**, whereas the 50% EtOH from *E. grandiflorus* (0.73 ± 0.01%) exhibited the highest concentration. Swertisin (**3**) was found in all extracts from both species, its highest content being measured in extracts of *E. grandiflorus* (70% EtOH, 3.70 ± 0.02%) and the lowest concentration in extracts prepared with DV2 of *E. macrophyllus* (70% EtOH, 0.08 ± 0.00%).

Chicoric acid (**4**) was quantified in all extracts prepared with 50% EtOH and 90% EtOH from DV1 of *E. macrophyllus* (50% EtOH, 3.50 ± 0.06%) and from *E. grandiflorus* (50% EtOH, 6.76 ± 0.12%); the last one showing a significantly higher concentration (*p* < 0.05), whereas the 50% EtOH extract from DvMus (0.57 ± 0.01%) showed the lowest content. Caffeoyl-feruloyl-tartaric acid (**5**) and di-feruloyl-tartaric acid (**6**) were found in all extracts, except **5** in the 96°GL EtOH extract of DVMus. The 50% EtOH extract of *E. grandiflorus* showed the highest contents of **5** (1.89 ± 0.04%) and **6** (1.13 ± 0.02%).

The quantification of chemical markers in the extracts of *E. macrophyllus* and *E. grandiflorus* did not provide sufficient differentiation between these species.

Up until now, the chemical composition of *E. grandiflorus* has been investigated more than *E. macrophyllus*; previous reports have included the quantitation of homoorientin by HPLC-DAD in 70% EtOH extracts [45], along with the determination of *cis*- and *trans*-aconitic acid and compounds **2**, **3**, and **4** in different extracts using a distinct HPLC-DAD method [12,13]. The *Brazilian Pharmacopoeia* [8] establishes caffeic acid, isoorientin, and swertiajaponin as chemical markers of *E. grandiflorus*. Among these markers, homoorientin was also described and quantified for *E. macrophyllus* and *E. grandiflorus* in this work. To the best of our knowledge, the quantitative chemical composition of *E. macrophyllus* extracts has not been reported so far, although some of the compounds herein quantified (**4**, **5**, and **6**) have been detected in this species, along with several other flavone C-glycosides and hydroxycinnamoyl tartaric acid derivatives [11,43].

### 3.4. In Vitro Inhibition of TNF Release

The potential anti-inflammatory activity of extracts from *E. macrophyllus* and *E. grandiflorus* leaves was assayed in vitro, by measuring their inhibitory effect on the release of TNF by LPS-stimulated THP-1 cells. The extracts were not cytotoxic for THP-1 cells at 60 µg/mL, showing cell viability above 90%, according to results from SRB and MTT assays. Furthermore, none of the extracts or dexamethasone (Dx) (used as a positive control) induced the release of TNF in the absence of the inflammatory stimulus (LPS), indicating an absence of a pro-inflammatory effect.

**Figure 3:**
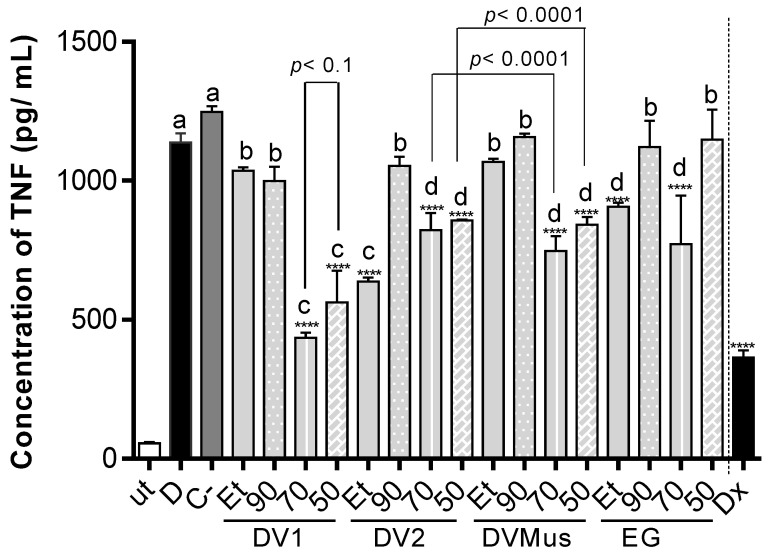
Effect on TNF release by LPS-stimulated THP-1 cells induced by extracts of leaves from *E. macrophyllus* prepared from different plant samples (DV1, DV2, and DVMus) and from a sample of *E. grandiflorus* (EG). The extracts were tested at 60 μg/mL. Data represent the mean ± SD of biological duplicates, with three experimental replicates each. **** *p* < 0.0001 compared with the challenged group. The significance level of the statistical test was set at *p* < 0.05. Ut—untreated cells; D—cells + LPS; C-—DMSO solvent (control); Dx—dexamethasone (0.2 µM); Et—96 °GL ethanol extract; 90—90% hydroethanolic extract; 70—70% hydroethanolic extract; 50—50% hydroethanolic extract. Different letters in the bars indicate significant differences.

The extracts prepared from the three samples of *E. macrophyllus* induced a similar inhibition of TNF release by LPS-stimulated THP-1 cells (Figure 3). None of the extracts obtained with 90% EtOH induced a significant reduction of this cytokine. Among the 96 °GL ethanol extracts, only those prepared from DV2 exhibited a significant reduction in TNF levels (44.1 ± 2.1%). All extracts prepared from *E. macrophyllus,* with either 70% EtOH or 50% EtOH, reduced the release of TNF significantly, reaching 61.9 ± 3.0% (70% EtOH extract) and 46.6 ± 10.2% (50% EtOH extract) inhibition for the extracts obtained from DV1. Furthermore, 70% EtOH and 50% EtOH extracts of DV2 induced TNF inhibition rates similar to the extracts prepared with DVMus (*p* > 0.05), whereas significant differences were obtained for those 70% EtOH and 50% EtOH extracts prepared from DV1 (*p* < 0.05). Taken together, the obtained results indicated that extracts prepared with 50% EtOH and 70% EtOH induced a more potent inhibition of TNF release for the three plant drugs of *E. macrophyllus*. It is worth noting that the 70% EtOH and 50% EtOH extracts of DV1 and DVMus were the most active extracts, showing similar activity without significant differences among them.

As for *E. grandiflorus*, the 96 °GL, 50%, and 70% EtOH extracts promoted significant reductions of TNF release by LPS-stimulated THP-1 cells (44.1 ± 2.1%, 20.4 ± 0.5%, and 32.2 ± 6.2%, respectively), whereas the 90% EtOH extract did show a less significant reduction of TNF.

We have previously investigated the effect of *E. grandiflorus* extracts on the release of TNF by LPS-stimulated THP-1 cells, non-differentiated into macrophages. In the previous study [12,13], it was observed that the 50% and 70% EtOH extracts exhibited significantly higher inhibition rates of TNF release (respectively, 100 ± 0.0% and 66.7 ± 5.0%) than those herein reported. Such differences may be related to the lower contents of flavone C-glycosides in the extracts here evaluated, since the authors ascribed the anti-inflammatory activity mainly to these compounds. Moreover, they did not employ cells differentiated into macrophages, as herein reported.

In general, the extracts showing high inhibitory activity of TNF release (70% EtOH and 50% EtOH) were those with the highest contents of aconitic acids and hydroxycinnamoyl tartaric acid derivatives. The data presented in this study supported the findings reported by [11], who observed a decrease in TNF and IL-1β levels in exudates when mice were treated with a fraction enriched in tartaric acid derivatives from *E. macrophyllus*, using an air pouch model of inflammation.

Data reported here for the in vitro inhibition of THF release by LPS-stimulated THP-1 cells treated with different extracts of *E. macrophyllus* and *E. grandiflorus* showed no significant differences in the responses induced by these species. Therefore, it was not possible to differentiate them based on the assayed biological activity, as similarly observed for the chemical markers quantified in the extracts.

### 3.5. In Vitro Anti-Oxidant Activity and Effect on ROS Production

The anti-oxidant activity of the extracts was assayed in vitro by the DPPH and β-carotene/linoleic acid co-oxidation radical scavenging methods. The extracts derived from *E. macrophyllus* DV1 exhibited significantly higher activity in the DPPH assay compared with those obtained from DV2 and DVMus. The EC_50_ values of the DV1 extracts were below 200 μg/mL, with the 50% EtOH extract demonstrating the highest activity (EC_50_ = 109.6 ± 1.7 μg/mL), as shown in Table 8. As for *E. grandiflorus*, only the 70% EtOH extract presented an EC_50_ value < 200 μg/mL (135.2 ± 2.0). The active extracts showed high contents of hydroxycinnamoyl tartaric acids (Table 7), suggesting the participation of these compounds in the observed anti-oxidant effect. In the same direction, *E. macrophyllus* fractions containing tartaric acid derivatives have been reported to promote significant reduction of DPPH radical by approximately 70% [11].

Regarding the β-carotene/linoleic acid co-oxidation assay, the most active extracts from *E. macrophyllus* were those prepared with 50% EtOH, either for DV1 (IC_50_ 40.1 ± 1.6 μg/mL), DV2 (IC_50_ 23.9 ± 1.8 μg/mL), or DVMus (IC_50_ 17.7 ± 1.5 μg/mL). In turn, all *E. grandiflorus* extracts were active, the 50% EtOH extract being the most active one (IC_50_ 6.8 ± 1.5 μg/mL) (Table 8). As far as we know, this is the first report on the anti-oxidant activity of extracts from *E. macrophyllus* and *E. grandiflorus* in the β-carotene/linoleic acid co-oxidation assay. Notably, among all extracts prepared with different plant drugs of both species, the 50% EtOH extracts showed the highest anti-oxidant activity, probably due to their higher contents of flavone-C-glycosides and hydroxycinnamoyl tartaric acid derivatives. This supposition was supported by several reports on the anti-oxidant action of different phenolic derivatives [46,47].

The results from the β-carotene/linoleic acid co-oxidation assay pointed out the 50% EtOH extracts as the most active ones and, for this reason, their effect on the ROS production by LPS-stimulated THP-1 cells was assayed. None of the assayed extracts inhibited ROS formation at high rates, with inhibition values below 20% (Table 8).

The anti-oxidant activity of a compound or extract can be more accurately estimated by using three experimental models that associate different oxidation mechanisms, including: (i) the formation, chain reactions, and action of free radicals; (ii) lipid peroxidation and degradation of defense cells systems; (iii) the formation of ROS [48]. In line with this recommendation, we utilized these different models to demonstrate the anti-oxidant potential of *E. macrophyllus* and *E. grandiflorus* extracts in our study.

Phenolic compounds are known to inhibit ROS due to the presence of hydroxyl groups, capable of scavenging free radicals by electron donation [49,50]. ROS inhibition can also result from the increase of cell anti-oxidant defenses [51]. Extracts of *Echinodorus* prepared with 50% EtOH promoted high DPPH radical capture, decreased lipid peroxidation, reduced ROS production, and diminished the release of TNF. These extracts possessed high contents of hydroxycinnamoyl tartaric acids and flavonoids, which may explain their anti-oxidant and anti-inflammatory action. These results demonstrated the potential of these extracts for further investigation as potential agents for the treatment of inflammatory disorders [52].

### 3.6. PCA Model

An exploratory unsupervised classification PCA model was built to investigate the similarity between *E. macrophyllus* and *E. grandiflorus*, based on the contents of chemical markers and biological activities of the assayed extracts. The extracts were allocated at the rows and the chemical markers (*cis*- and *trans*-aconitic acids, CTAA; homoorientin, HOMO; swertisin, SWER; chicoric acid, CHA; caffeoyl-feruloyl-tartaric acid, CATA; diferuloyl-tartaric acid, DFTA) were put in the columns, along with data from the biological assays (lipid peroxidation in the β-carotene assay, Bcar; DPPH radical scavenging, DPPH; inhibition of TNF release, TNF), generating a matrix of dimensions 144 × 11. Data were preprocessed by autoscaling to provide equal weights for all investigated variables in both methods [15].

**Figure 4:**
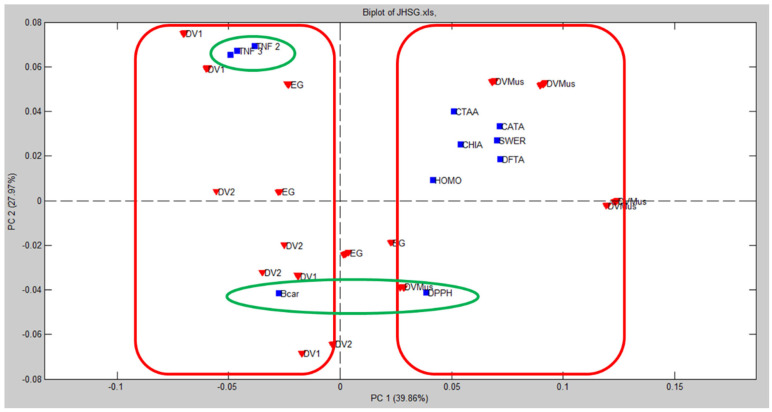
Biplot provided by a PCA model built for *E. macrophyllus* and *E. grandifloras* samples based on the quantitative chemical composition and in vitro biological activity of extracts. Red down triangles represent scores/samples and blue squares represent loadings/variables.

The obtained results for PCA model are depicted in Figure 4 as a biplot of PC1 × PC2. A biplot is a graphical representation simultaneously showing the relations among scores (describing samples) and loadings (describing variables). A model with two PCs accounted for 67.83% of the total variance data. In this model, none of the samples presented Q residues above the limit at a 95% confidence level, thus indicating the absence of outliers in the dataset. PC1 accounted for 39.86% of variance and discriminated DVMus (positive scores) from DV1/DV2 (negative scores) samples (these two clusters are indicated by red rectangles in Figure 4). EG samples were not clearly discriminated by this PCA model, even when PC3 was considered (10.8% of the variance), and it was not possible to ascribe its similarity to any of the evaluated samples of *E. macrophyllus*. Based on the PC1 of this model, we could conclude that the variation within different samples of the same species (DV, *E. macrophyllus*) was greater than the variation between the two species. In other words, the differences observed within *E. macrophyllus* samples were more pronounced than the differences between *E. macrophyllus* and *E. grandiflorus*. In addition, the anti-inflammatory (TNF) and anti-oxidant (DPPH/Bcar) activities were captured by PC2 (27.97%), respectively, with positive and negative loadings (green circles).

**Figure 5:**
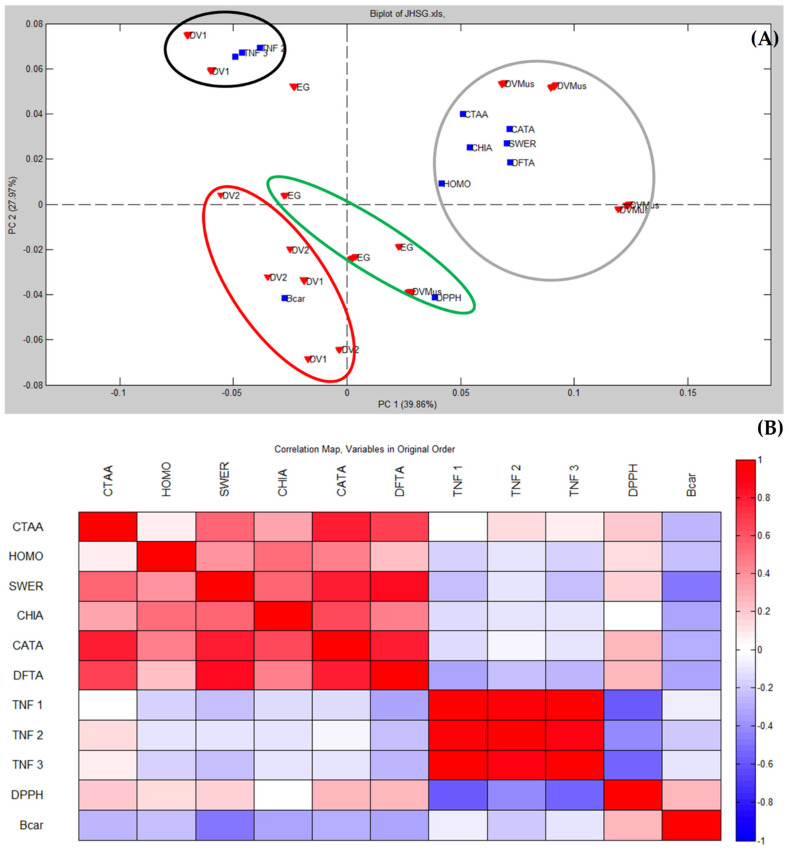
(**A**) The same PCA biplot of Figure 4 showing variable clusters: red down triangles represent scores and blue squares represent loadings. (**B**) Heatmap of chemical markers’ contents and biological activity. CATA—*cis* and *trans*-aconitic acids; HOMO—homoorientin; SWER—swertisin; CHIA—chicoric acid; CATA—caffeoyl-feruloyl-tartaric acid; TNF 1, 2, and 3 (inhibition of TNF release); Bcar—lipid peroxidation in β-carotene assay; DPPH—DPPH radical scavenging assay.

In Figure 4, samples were clustered as a function of the scores/samples in PC1. Aiming to better visualize interrelations, Figure 5A shows the same PCA biplot of Figure 4, but with marked clusters as a function of the loadings/variables. As depicted in Figure 5, the inhibition of TNF release was related to DV1, suggesting a positive correlation between these variables (see the black ellipse). Moreover, the anti-oxidant activity Bcar was associated to DV2 (red ellipse), while the DPPH activity was weakly correlated to EG (green ellipse). Chemical markers (CTAA, HOMO, SWER, CHA, CATA, and DFTA) were positively correlated to DVMus (grey circle in Figure 5A), which was consistent with the higher concentrations of these assayed constituents in this sample.

A heatmap (correlation map) was also generated to characterize the relationship between the analyzed variables (Figure 5B). The inhibition of TNF release showed weak correlations with CTAA (*r_TNF-1_* = 0.0198; *r_TNF-2_* = 0.1543; *r_TNF-3_* = 0.0732), which, in turn, correlated moderately with DPPH activity (*r =* 0.2149). DPPH activity showed weak/moderate correlations to all assayed chemical markers: HOMO (*r =* 0.1545), SWER (*r =* 0.1654), CHIA (*r =* 0.303), CATA (*r =* 0.2755), and DFTA (*r =* 0.2507). Conversely, these chemical markers were inversely correlated to Bcar anti-oxidant activity: CTAA, *r =* −0.2709; HOMO, *r =* −0.2344; SWER, *r =* −0.4801; CHIA, *r =* −0.3293; CATA, *r =* −0.2847; DFTA, *r =* −0.3321. The apparent contradictory results observed in the anti-oxidant assays could be attributed to the differences in the methods employed. The DPPH assay was based on the measurement of radical scavenging activity, while the β-carotene test evaluated the inhibition of lipid peroxidation. These assays targeted different mechanisms of anti-oxidant activity and may have elicited distinct responses from the constituents present in the extracts [53].

The inhibition of lipid peroxidation is regarded as a more complex and selective method for identifying anti-oxidant compounds than DPPH, although both methods are important to characterize the anti-oxidant response [54]. The DPPH assay has been known to exhibit nonspecific responses to various compounds and may yield misleading results when used in isolation. To ensure a more accurate assessment of anti-oxidant activity, it is recommended to analyze the DPPH assay in conjunction with other complementary anti-oxidant tests [55]. The literature data have indicated low correlation between results from DPPH test and other anti-oxidant assays [56], as herein observed for the low positive correlation with the β-carotene assay (*r* = 0.2690).

PCA results clearly demonstrated the similarities in the quantitative chemical composition and biological activities of *E. macrophyllus* and *E. grandiflorus* extracts, which was in accordance with previous reports [57]. However, PCA was not able to discriminate *E. grandiflorus* from the analyzed samples of *E. macrophyllus.* The analyzed chemical markers showed a stronger correlation with DVMus, allowing for the differentiation between *E. macrophyllus* samples (DVMus vs. DV1 and DV2). However, these same chemical markers, which had positive loadings on PC1, did not show a significant correlation with *E. grandiflorus* extracts. Therefore, based on the quantification of these chemical markers and the assessed biological activities in this study, it was suggested that *E. macrophyllus* and *E. grandiflorus* could not be distinguished from each other.

Furthermore, our findings indicated that the two commercial lots of *E. macrophyllus* (DV1 and DV2) exhibited comparable contents of the analyzed chemical markers and presented similar biological activities. However, according to the PCA model, DVMus displayed distinct chemical marker contents compared with DV1 and DV2. This divergence could be attributed to the fact that DVMus was sourced from a single cultivated specimen, while DV1 and DV2 were composed of multiple specimens grown in different environments. These varying growth conditions could impact the chemical profiles of the plants [58].

## 4. Conclusions

Our study explored, for the first time, molecular analysis, quantitative chemical composition, and biological activity of various extracts from *E. macrophyllus* and *E. grandiflorus* leaves aiming to highlight the similarities and differences between these species. The molecular analysis of *E. macrophyllus* did not allow its genetic differentiation from other *Echinodorus* species, including *E. grandiflorus*, suggesting that there are only a few distinct genetic characteristics separating these species. The quantitation of six chemical markers in extracts prepared from commercial samples (DV1 and DV2) and a cultivated specimen (DVMus) of *E. macrophyllus*, along with a commercial lot of *E. grandiflorus* (EG), revealed higher contents of these markers in the 50% and 70% ethanol extracts. However, we were unable to differentiate between the species based on the concentrations of these markers. Moreover, the 50% and 70% ethanol extracts also elicited higher biological activity—inhibition of TNF release by LPS-stimulated THP-1 cells and anti-oxidant activity—in comparison with the 90% and 96°GL ethanol extracts. This difference in activity was attributed to the higher contents of chemical markers in the former extracts. Nevertheless, the results from the biological assays did not allow us to discriminate between the two species. Overall, our data demonstrated that *E. macrophyllus* and *E. grandiflorus* have similar chemical composition and biological activity. Additionally, the PCA analysis revealed a comparable chemical composition and biological activity among the commercial samples of *E. macrophyllus*. However, the analysis successfully distinguished the chemical composition of the cultivated specimen from the commercial lots. Interestingly, the cultivated and commercial samples of *E. macrophyllus* exhibited a higher level of intra-specific variance compared with the inter-specific variance observed with *E. grandiflorus*. It is worth noting, though, that the quantified chemical markers and biological activities assessed in this study did not reveal any discernible differences between the two *Echinodorus* species.

As part of our future perspectives, we plan to conduct in vivo studies to investigate the anti-inflammatory activity of the 50% and 70% ethanol extracts from *E. macrophyllus*. These studies will involve the use of proof-of-concept models as well as disease models, including acute and chronic antigen-induced arthritis. By evaluating the efficacy of these extracts in vivo, we aim to contribute to the development of an herbal preparation from *E. macrophyllus* that can be potentially utilized for managing arthritis and other inflammatory diseases. These studies will provide valuable insights into the therapeutic potential of *E. macrophyllus* and its application in the field of inflammatory disorders.

## Figures and Tables

**Table 1:** Best matches between the sequences of *Echinodorus macrophyllus* (MHNJB) and the GenBank database using blastn.

Marker	Best Matches	% Identity	Identities bp	Query Cover (%)	Accession No.
ITS	*Echinodorus osiris*	99.56	684/687	100	KT437651
	*Echinodorus longiscapus*	99.42	683/687	100	KT437653
	*Echinodorus portoalegrensis*	99.42	683/687	100	KT437648
	*Echinodorus barthii*	99.27	682/687	100	KT437652
	*Echinodorus opacus*	99.13	681/687	100	KT437650
*psbA-trnH*	*Echinodorus paniculatus*	100.00	463/463	100	HM367285
	*Echinodorus grandiflorus*	99.78	462/463	100	HM367294
	*Echinodorus longiscapus*	99.78	462/463	100	HM367292
	*Echinodorus muricatus*	99.57	462/464	100	DQ786514
	*Echinodorus cordifolius*	99.35	460/463	100	HM367302
*matK-trnK*	*Echinodorus paniculatus*	99.85	1309/1311	100	OK587808
	*Echinodorus cordifolius*	99.85	1309/1311	100	OK587807
	*Echinodorus paniculatus*	99.85	1309/1311	100	EF088097
	*Echinodorus longiscapus*	99.77	1308/1311	100	EF088112
	*Echinodorus grandiflorus*	99.69	1307/1311	100	EF088113
Leafy	*Echinodorus cordifolius*	99.07	212/214	100	HM367210
	*Echinodorus paniculatus*	99.07	212/214	100	EF088144
	*Echinodorus uruguayensis*	98.60	211/214	100	EF088159
	*Echinodorus cordifolius*	98.60	211/214	100	EF088172
	*Echinodorus bracteatus*	98.60	212/215	100	EF088170

**Table 2:** Characterization of the markers used in the phylogenetic analyses. VI—number of variable informative characters; CI—consistency index; RI—retention index; hLRTs—hierarchical likelihood ratio tests.

Marker	Terminals	Characters	VI (%)	CI	RI	Model (hLRTs)
ITS	37	725	100 (13.8%)	0.71	0.8	GTR+G
5S-NTS	28	266	98 (36.8%)	0.68	0.7	HKY+G
LEAFY	43	306	33 (10.8%)	0.93	0.95	K80+G
*matK-trnK*	35	1311	164 (12.5%)	0.86	0.92	GTR+G
*psbA-trnH*	46	574	108 (18.8%)	0.81	0.91	GTR+G
Combined	47	3182	503 (15.8%)	0.73	0.82	

**Table 3:** Mean pairwise distances within and between the major clades recovered in the phylogenetic analysis. Mean distances within groups are highlighted in bold. N indicates the number of terminals in each group. n/c denotes cases in which it was not possible to estimate evolutionary distance.

	N	1	2	3	4	5	6	7	8
1. Clade F	30	**0.007**							
2. *E. glaucus*	1	0.029	**n/c**						
3. Clade E	3	0.051	0.052	**0.026**					
4. *E. bracteatus*	1	0.042	0.050	0.046	**n/c**				
5. Clade D	2	0.071	0.073	0.073	0.068	**0.001**			
6. Clade B	6	0.065	0.067	0.065	0.059	0.078	**0.022**		
7. Clade A	3	0.094	0.089	0.090	0.090	0.095	0.092	**0.037**	
8. *E. berteroi*	1	0.109	0.112	0.109	0.107	0.121	0.111	0.134	**n/c**

**Table 4:** Mean pairwise distances for each marker within the major clades recovered in the phylogenetic analysis.

	ITS	5S-NTS	LEAFY	*matK-trnK*	*psbA-trnH*	Combined
Clade F	0.008	0.062	0.015	0.003	0.004	0.007
Clade E	0.033	0.067	0.047	0.012	0.025	0.026
Clade D	0.003	0.004	0.000	0.000	0.000	0.001
Clade B	0.043	0.167	0.019	0.008	0.015	0.022
Clade A	0.019	0.127	0.029	0.025	0.058	0.037

**Table 5:** Results of system suitability tests for chromatographic determination of compounds **1**–**6** from *E. macrophyllus*.

Compound	Retention Factor (*k*)	USP Tailing Factor	Number of Theoretical Plates (N)	RSD Values of Retention Time (%)	Resolution (Rs)
*cis*- and *trans*-aconitic acid (**1**)	0.49	1.07	5656	0.12	1.87
homoorientin (**2**)	4.49	1.29	7480	0.07	3.41
swertisin (**3**)	7.20	1.02	6441	0.40	1.50
chicoric acid (**4**)	9.40	1.19	6756	0.08	1.55
caffeoyl-feruloyl-tartaric acid (**5**)	13.38	1.13	2147	0.22	2.65
di-feruloyl-tartaric acid (**6**)	15.42	1.05	6857	0.11	2.00

**Table 6:** Calibration results, LOQ, LOD, precision of the intra-day (*n* = 6) and inter-day (*n* = 12) assays, and recovery measurements of constituents quantified by UPLC-DAD in *E. grandiflorus* and *E. macrophyllus*.

Compound	Regression Equation	Linear Range (μg)	*r* ^2^	LOQ (μg)	LOD (μg)	Intra-Day RSD	Inter-Day RSD	Recovery (% ± SD)
**1**	*y* = 43,603*x* − 19,041	6.25–100	0.9999	0.990	0.300	2.65	2.60	96.0 ± 3.9 110.8 ± 4.8 100.5 ± 3.8
**2**	*y* = 38,042*x* − 14,758	1.25–20	0.9998	0.216	0.065	0.23	1.55	110.2 ± 0.8 95.8 ± 4.4 98.8 ± 2.1
**3**	*y* = 9441*x* + 23,371	3.0–150	0.9969	1.928	0.584	4.90	4.27	99.5 ± 1.9 107.9 ± 1.1 100.4 ± 2.9
**4**	*y* = 47,326*x* − 53,912	9.37–300	0.9993	0.048	0.014	0.84	1.79	99.9 ± 0.7 100.0 ± 1.1 98.9 ± 2.1

**Table 7:** Extractive yields and concentrations (mean ± SD of triplicates) of chemical markers in extracts of *E. macrophyllus* and *E. grandiflorus*.

Concentration (% *w*/*w*)								
Plant Material	Extract	Extractive Yield (% *w*/*w*)	*cis*- and *trans*- Aconitic Acid (1)	Homoorientin (2)	Swertisin (3)	Chicoric Acid (4)	Caffeoyl-Feruloyl-Tartaric Acid (5)	Di-Feruloyl-Tartaric Acid (6)
DV1	96 °GL EtOH	8.51	0.491 ± 0.012 ^a^	0.052 ± 0.001 ^a^	0.171 ± 0.002 ^a^	0.750 ± 0.013 ^a^	0.636 ± 0.003 ^a^	0.630 ± 0.005 ^a^
	90% EtOH	9.98	2.992 ± 0.036 ^b^	0.095 ± 0.002 ^b^	0.260 ± 0.013 ^b^	1.531 ± 0.012 ^b^	0.794 ± 0.002 ^b^	0.751 ± 0.006 ^b^
	70% EtOH	21.31	8.094 ± 0.024 ^c^	0.164 ± 0.007 ^b^	0.382 ± 0.009 ^c^	2.853 ± 0.042 ^c^	0.991 ± 0.007 ^c^	0.862 ± 0.008 ^c^
	50% EtOH	25.34	10.333 ± 0.082 ^d^	0.190 ± 0.006 ^c^	0.480 ± 0.011 ^d^	3.505 ± 0.059 ^d^	1.081 ± 0.008 ^c^	0.913 ± 0.003 ^c^
DV2	96 °GL EtOH	11.14	ND	0.140 ± 0.002 ^b^	0.164 ± 0.004 ^a^	0.577 ± 0.002 ^e^	0.570 ± 0.001 ^a^	0.595 ± 0.001 ^a^
	90% EtOH	14.40	2.311 ± 0.106 ^e^	0.261 ± 0.003 ^d^	0.743 ± 0.015 ^e^	2.408 ± 0.060 ^c^	0.798 ± 0.007 ^b^	0.944 ± 0.005 ^c^
	70% EtOH	19.13	1.263 ± 0.036 ^f^	0.141 ± 0.001 ^b^	0.082 ± 0.004 ^f^	0.740 ± 0.005 ^a^	0.992 ± 0.003 ^c^	0.743 ± 0.004 ^b^
	50% EtOH	22.58	1.244 ± 0.020 ^f^	0.083 ± 0.009 ^a^	0.172 ± 0.004 ^a^	0.572 ± 0.001 ^e^	1.089 ± 0.009 ^c^	0.690 ±0.004 ^b^
DVMus	96 °GL EtOH	4.07	ND	ND	0.181 ± 0.004 ^a^	ND	ND	0.578 ± 0.010 ^a^
	90% EtOH	19.65	0.762 ± 0.030 ^g^	0.046 ± 0.008 ^a^	0.312 ± 0.015 ^c^	0.612 ± 0.005 ^e^	0.581 ± 0.018 ^a^	0.607 ± 0.008 ^a^
	70% EtOH	21.51	7.473 ± 0.027 ^h^	0.020 ± 0.004 ^a^	0.235 ± 0.005 ^b^	ND	0.574 ± 0.004 ^a^	0.585 ± 0.012 ^a^
	50% EtOH	22.21	7.595 ± 0.080 ^h^	ND	0.244 ± 0.007 ^b^	0.576 ± 0.001 ^e^	0.574 ± 0.001 ^a^	0.581 ± 0,013 ^a^
EG	96 °GL EtOH	2.97	ND	0.130 ± 0.001 ^a^	1.432 ± 0.063 ^g^	0.718 ± 0.005 ^a^	0.620 ± 0.002 ^a^	0.630 ± 0.034 ^a^
	90% EtOH	11.37	ND	0.262 ± 0.005 ^d^	2.742 ± 0.130 ^h^	1.320 ± 0.050 ^b^	0.852 ± 0.017 ^b^	0.831 ± 0.026 ^c^
	70% EtOH	22.13	1.731 ± 0.063 ^i^	0.352 ± 0.006 ^e^	3.701 ± 0.022 ^i^	4.471 ± 0.087 ^f^	1.262 ± 0.008 ^c^	0.852 ± 0.014 ^c^
	50% EtOH	22.76	20.123 ± 0.161 ^j^	0.734 ± 0.007 ^f^	0.452 ± 0.017 ^d^	6.762 ± 0.118 ^g^	1.893± 0.005 ^c^	1.134 ± 0.017 ^d^

Legend: SD—standard deviation; DV1—*E. macrophyllus* commercial sample 1; DV2—*E. macrophyllus* commercial sample 2; DVMus—*E. macrophyllus* UFMG sample; EG—*E. grandiflorus* commercial sample; EtOH—96 °GL ethanol extract; 90% EtOH—90% hydroethanolic extract; 70% EtOH—70% hydroethanolic extract; 50% EtOH—50% hydroethanolic extract. Differences between the mean concentrations of the extracts were analyzed by ANOVA, followed by Tukey test (*p* < 0.05). For the contents of chemical markers, different letters within the columns indicate significant differences. ND—not determined (bellow the LOQ).

**Table 8:** Anti-oxidant activity of extracts of *E. macrophyllus* (DV1, DV2, and DVMus) and *E. grandiflorus* assayed in vitro by the DPPH and β-carotene/linoleic acid co-oxidation models and effect of selected extracts on ROS produced by LPS-stimulated THP-1 cells.

Plant Drug	Extract	DPPH (EC_50_ μg/mL ± SD)	β-Carotene (IC_50_ μg/mL ± SD)	ROS (% Reduction)
DV1	96 °GL EtOH	>200 ^a^	>200 ^a^	NA
	90% EtOH	140.2 ± 1.5 ^b^	44.4 ± 1.6 ^b^	NA
	70% EtOH	139.2 ± 1.8 ^b^	41.7 ± 1.5 ^b^	NA
	50% EtOH	109.6 ± 1.7 ^c^	40.1 ± 1.6 ^b^	9.1 ± 5.5 ^a^
DV2	96 °GL EtOH	>200 ^a^	>200 ^a^	NA
	90% EtOH	>200 ^a^	>200 ^a^	NA
	70% EtOH	>200 ^a^	>200 ^a^	NA
	50% EtOH	>200 ^a^	23.9 ± 1.8 ^c^	14.8 ± 3.4 ^b^
DVMus	96 °GL EtOH	180.8 ± 1.5 ^d^	22.6 ±1.6 ^c^	NA
	90% EtOH	>200 ^a^	36.0 ± 1.9 ^b^	NA
	70% EtOH	>200 ^a^	32.8 ± 1.5 ^b^	NA
	50% EtOH	>200 ^a^	17.7 ± 1.5 ^d^	16.3 ± 2.2 ^c^
EG	96 °GL EtOH	>200 ^a^	8.7 ±1.5 ^e^	NA
	90% EtOH	>200 ^a^	11.9 ± 1.4 ^f^	NA
	70% EtOH	135.2 ± 1.9 ^b^	8.3 ± 1.1 ^e^	NA
	50% EtOH	>200 ^a^	6.8 ± 1.5 ^g^	19.0 ± 6.2 ^d^

Legend: DPPH—2,2-diphenyl-1-picrylhydrazyl radical; NA—not analyzed; IC_50_—50% inhibitory concentration; EC_50_—50% effective concentration; SD—standard deviation; DV1—*E. macrophyllus* commercial sample 1; DV2—*E. macrophyllus* commercial sample 2; DVMus—*E. macrophyllus* UFMG sample; EG—*E. grandiflorus* commercial sample; EtOH—96 °GL ethanol extract; 90% EtOH—90% hydroethanolic extract; 70% EtOH—70% hydroethanolic extract; 50% EtOH—50% hydroethanolic extract. Differences between the mean concentrations of the extracts were analyzed by ANOVA, followed by Tukey test (*p* < 0.05). Different letters within the columns indicate significant differences.

## Data Availability

Data is contained within the article or supplementary material.

## Supplementary Materials

The following supporting information can be downloaded at: https://www.mdpi.com/article/10.3390/antiox12071365/s1, Table S1: Primers used in PCR and sequencing; Table S2: GenBank accessions and voucher data of the terminals used in the phylogenetic analyses of Echinodorus, Supplementary References are [59,60,61,62,63,64].
