# Supplementary material for: Quantitative Chemical Composition, Anti-Oxidant Activity, and Inhibition of TNF Release by THP-1 Cells Induced by Extracts of Echinodorus macrophyllus and Echinodorus grandiflorus
Source: Antioxidants (Basel). 2023 Jun 29;12(7):1365. doi: 10.3390/antiox12071365 (PMC10376401; doi:10.3390/antiox12071365)
Supplement: Supplementary file 1 [file antioxidants-12-01365-s001.zip › antioxidants-2430357-supplementary.pdf]

Supplementary Material

Quantitative chemical composition, antioxidant activity and inhibition of TNF release by THP-1 cells induced by extracts of *Echinodorus macrophyllus* and *Echinodorus grandiflorus*

Marina Pereira Rocha[1], Lyandra Maciel Cabral da Silva[1], Laura Paulino Maia Silva[1], José Hugo de Sousa Gomes[1], Rodrigo Maia de Pádua[1], João Aguiar Nogueira Batista[2], Marcelo Martins Sena[3,4], Priscilla Rodrigues Valadares Campana[1], Fernão Castro Braga[1*]

[1]Departament of Pharmaceutical Sciences, Faculty of Pharmacy, Universidade Federal de Minas Gerais, campus Pampulha, Avenida Antônio Carlos 6627, 31.270-901, Belo Horizonte, MG, Brazil

[2]Departament of Botany and Molecular Biology, Universidade Federal de Minas Gerais, campus Pampulha, Avenida Antônio Carlos 6627, 31.270-901, Belo Horizonte, MG, Brazil

[3]Chemistry Department, Universidade Federal de Minas Gerais, Av. Antônio Carlos 6627, Belo Horizonte, Brazil

[4]Instituto Nacional de Ciência e Tecnologia em Bioanalítica (INCT-Bio), Campinas, SP 13083-970, Brazil

Supplementary Table S1. Primers used in PCR and sequencing.

Marker	Primer	Sequence 5' → 3'	TM	Reference
ITS	ITS4	TCCTCCGCTTATTGATATGC	54°C	White et al. 1990
	ITS5	GGAAGTAAAAGTCGTAACAAGG	53°C	
	ITS-ER	ATGCTTAAACTCRGCGGGTGA	59/62°C	
5S-NTS	PI	TGGGAAGTCCTYGTGTTGCA	59/62°C	Cox et al. 1992
	PII	KTMGYGCTGGTATGATCGCA	56/63°C	
Leafy	FLint2-F1	CTTCCACCTCTACGACCAGTG	60°C	Grob et al. 2004
	FLint2-R1	TCTTGGGCTTGTTGATGTAGC	58°C	
matK	matK-EF	GAAGAATTCCAAAARGATTT	45/47°C	Liang & Hilu 1996
	matK-ER	TCCTTGATATCGAACATAATG	49°C	
	MG1	CTACTGCAGAACTAGTCGGATGGAGTA GAT	63°C	
	MG15	ATCTGGGTTGCTAACTCAATG	55°C	
psbA-trnH	psbA	GTTATGCATGAACGTAATGCTC	54°C	Sang et al. 1997
	trnH2	CGCGCATGGTGGATTCACAATCC	64°C	Tate & Simpson 2003

References

Cox, A.V., Bennet, M.D., Dyer, T.A., 1992. Use of the polymerase chain reaction to detect spacer size heterogeneity in plant 5S-rRNA gene clusters and to locate such clusters in wheat (*Triticum aestivum* L.). Theor. Appl. Genet. 83, 684–690.

Grob, G.B.J., Gravendeel, B., Eurlings, M.C.M., 2004. Potential phylogenetic utility of the nuclear FLORICAULA/LEAFY second intron: comparison with three cloroplast DNA regions in *Amorphophallus* (Araceae). Mol. Phylogenet. Evol. 30, 13–23.

Liang, H., Hilu, K.W., 1996. Application of matK gene sequences to grass systematics. Can. J. Bot. 74, 125–134.

Sang, T, Crawford, D. J. and Stuessy, T. F. (1997). Chloroplast DNA phylogeny, reticulate evolution, and biogeography of *Paeonia* (Paeoniaceae). American Journal of Botany, 84, 1120-1136.

Tate, J. A. and Simpson, B. B. (2003). Paraphyly of *Tarasa* (Malvaceae) and diverse origins of the polyploid species. Systematic Botany, 28, 723-737.

White, T.J., Bruns, T., Lee, S., Taylor, J., 1990. Amplification and direct sequencing of fungal ribosomal RNA genes for phylogenetics. In: Innis, M.A., Gelfund, D.H., Sninsky, J.J., White, T.J. (Eds.), PCR Protocols: a Guide to Methods and Applications. Academic Press, San Diego, pp. 315–32.

Supplementary Table S2. GenBank accessions and voucher data of the terminals used in the phylogenetic analyses of *Echinodorus*.

Taxon	Voucher	ITS	LEAFY	*psbA-trnH*	*matK-trnK*	5S
Echinodorus africanus Rataj	PR745390	KT437644	-	-	-	-
Echinodorus barthii H.Muehlberg	Lehtonen 702 (TUR)	KT437652	HM367221	HM367327	KT437639	-
Echinodorus berteroi (Spreng.) Fassett	Lehtonen & Ramirez-Garcia 412 (TUR)	EF088087	EF088181	HM367270	EF088134	EF088022
Echinodorus bracteatus Micheli	Lehtonen & Navarrete 491 (TUR)	EF088076	EF088170	HM367301	EF088124	EF088040
Echinodorus opacus Rataj	Bogner s.n. (TUR)	KT437650	KT437657	KT437643	KT437634	-
Echinodorus cf. osiris Rataj	Quester s.n. (TUR)	EF088094	EF088188	HM367324	-	EF088042
Echinodorus cf. opacus Rataj	Quester s.n. (TUR)	-	HM367214	HM367323	-	-
Echinodorus cf. opacus Rataj	Bogner s.n.	-	HM367214	HM367323	KT437634	-
Echinodorus cordifolius (L.) Griseb.	Lehtonen 457 (TUR)	EF088078	EF088172	HM367302	EF088126	EF088027
Echinodorus cordifolius (L.) Griseb.	Lehtonen & Ramirez-Garcia 417 (TUR)	EF088079	EF088173	HM367303	EF088127	EF088028
Echinodorus decumbens Kasselm.	Lehtonen 392 (TUR)	EF088069	EF088163	HM367296	EF088117	EF088018
Echinodorus emersus Lehtonen	Lehtonen 140 (TUR)	EF088051	EF088147	HM367286	EF088100	EF088001

Echinodorus floribundus (Seub.) Seub.	Lehtonen 188 (TUR)	EF088054	EF088150	HM367287	EF088103	EF088004
Echinodorus gabrielii Rataj	Bogner s.n. (TUR)	KT437647	HM367225	HM367329	KT437633	-
Echinodorus glaucus Rataj	Muhlberg s.n. (TUR)	EF088084	EF088178	HM367307	EF088132	EF088033
Echinodorus grandiflorus (Cham. & Schltdl.) Micheli	Lehtonen 393 (TUR)	EF088070	EF088164	HM367297	EF088118	EF088019
Echinodorus grandiflorus (Cham. & Schltdl.) Micheli	Lehtonen 391 (TUR)	EF088065	HM367239	HM367337	EF088113	EF088014
Echinodorus grisebachii **Rataj** Small	Lehtonen 151 (TUR)	EF088053	EF088149	HM367272	EF088102	EF088003
Echinodorus heikobleheri Rataj	Quester s.n. (TUR)	EF088093	EF088187	HM367274	EF088139	EF088041
Echinodorus horemanii Rataj	Falck 96 (TUR)	-	HM367230	HM367332	-	-
Echinodorus horizontalis Rataj	Lehtonen & Rodriguez Arevalo 99 (TUR)	EF088047	EF088143	HM367282	EF088096	EF087997
Echinodorus inpai Rataj	Muhlberg s.n. (TUR)	EF088092	EF088186	HM367310	EF088138	-
Echinodorus longipetalus Micheli	Lehtonen & Burguez 271 (TUR)	EF088067	EF088162	HM367284	EF088115	EF088016
Echinodorus longiscapus Arechav.	Lehtonen & Delfino 341 (TUR)	EF088063	EF088158	HM367292	EF088112	EF088012
Echinodorus longiscapus Arechav.	Lehtonen & Dematteis 204 (TUR)	EF088059	HM367245	HM367289	EF088108	EF088009
Echinodorus macrophyllus	Lehtonen 700 (TUR)	-	HM367206	HM367314	-	-

(Kunth) Micheli						
Echinodorus maculatus Somogyi	Lehtonen 719 (TUR)	KT437645	HM367238	HM367336	KT437635	-
Echinodorus muricatus Griseb.	Botanic Garden, Natural History Museum, UiO, #1993-30	-	-	DQ786514	-	-
Echinodorus Osiris Rataj	Lehtonen 698 (TUR)	KT437651	HM367222	HM367328	KF632793	-
Echinodorus paniculatus Micheli	Lehtonen & Pacheco 469 (TUR)	EF088082	EF088176	HM367306	EF088130	EF088031
Echinodorus paniculatus Micheli	Lehtonen 168 (TUR)	EF088048	EF088144	HM367285	EF088097	EF087998
Echinodorus portoalegrensis Rataj	TUR:Bogner s.n.	KT437648	HM367226	HM367330	KT437632	-
Echinodorus pseudohorizontalis Rataj	PR:PR745393	-	KT437641	-	-	-
Echinodorus pubescens Rataj	Harley et al. 20019 (AAU)	-	EF088193	HM367312	-	EF088045
Echinodorus reptilis Lehtonen	Lehtonen & Burguez 261 (TUR)	EF088061	EF088156	HM367290	EF088110	EF088010
Echinodorus scaber Rataj	Lehtonen & Pacheco 440 (TUR)	EF088080	EF088174	HM367304	EF088128	EF088029
Echinodorus subalatus Mart. ex Schult.f.) Griseb.	Lehtonen & Pacheco 472 (TUR)	EF088075	EF088169	HM367300	EF088123	EF088024
Echinodorus subalatus Mart. ex Schult.f.) Griseb.	Lehtonen 394 (TUR)	EF088071	EF088165	HM367298	EF088119	EF088020
Echinodorus trialatus Fassett	Lehtonen & Pacheco 441 (TUR)	EF088074	EF088168	HM367299	EF088122	EF088023
Echinodorus tunicatus Small	Lehtonen 133 (TUR)	EF088058	EF088154	HM367283	EF088107	EF088008

Echinodorus uruguayensis Arechav.	Lehtonen et al. 237(TUR)	EF088066	EF088161	HM367295	EF088114	EF088015
Echinodorus viridis Rataj	PR:PR745396	-	-	KT437642	-	-
Echinodorus sp.	Lehtonen & Burguez 275 (TUR)	EF088062	EF088157	HM367291	EF088111	EF088011
Echinodorus sp.	Lehtonen & Burguez 309 (TUR)	EF088085	EF088179	HM367308	EF088133	EF088034
